# The European Innovation Partnership on Active and Healthy Ageing Synergies: Protocol for a Prospective Observational Study to Measure the Impact of a Community-Based Program on Prevention and Mitigation of Frailty (ICP – PMF) in Community-Dwelling Older Adults

**Published:** 2016-11-01

**Authors:** G Liotta, F Orfila, M Vollenbroek-Hutten, R Roller-Winsberger, M Illario, D Musian, S Alvino, R O’Caoimh, A Cano, W Molloy, G Iaccarino, MC Marazzi, MC Inzerilli, O Madaro, C Paul, P Csonka, AC Vince, E Menditto, M Maggio, P Scarcella, F Gilardi, F Lucaroni, P Abete, V Girardi, R Barra, L Palombi

**Affiliations:** 1 Biomedicine and Prevention Dept. – University of Rome Tor Vergata, Rome, Italy; 2 Institut Universitari d’Investigació en Atenció Primària Jordi Gol (IDIAP Jordi Gol), Barcelona, Spain; 3 Faculty of Electrical Engineering, Mathematics and Computer Science, Telemedicine group, University of Twente, Enschede, The Netherlands; 4 Ziekenhuis Groep Twente, Almelo, The Netherlands; 5 Medical University of Graz, Austria; 6 DISMET, Federico II University Naples, Italy; 7 SI4Life, Genoa, Italy; 8 Health Research Board, Clinical Research Facility Galway, National University of Ireland, Galway, Ireland; 9 Department of Pediatrics, Obstetrics and Gynecology, University of Valencia, Spain; 10 Centre for Gerontology and Rehabilitation, University College Cork, St Finbarrs Hospital, Cork City, Ireland; 11 Department of Medicine, University of Salerno, Baronissi, Salerno; 12 LUMSA University, Rome, Italy; 13 Community of Sant’Egidio - Long Live the Elderly program, Rome Italy; 14 Rome Municipality, Italy; 15 ICBAS, University of Porto, Portugal; 16 Educators’ Centre Association, Pecs, Hungary; 17 CIRFF, Center of Pharmacoeconomics, University of Naples Federico II 17University of Parma, Italy; 18 University-Hospital of Parma, Emilia Romagna Region Italy; 19 Associazione Salute in Collina, Naples, Italy

**Keywords:** frailty, community-based programs, hospitalization, institutionalization, death rate

## Abstract

Aim of this paper is to describe the protocol of the study “Impact of a Community-based Program on Prevention and Mitigation of Frailty in community-dwelling older adults” developed in the framework of the European Innovation Partnership on Active and Healthy Ageing. This proposal has been developed by the Partnership Action groups on frailty, fall prevention and polypharmacy in older. The proposal wants to assess the impact of community-based programs aimed to counteract three main outcomes related to frailty: hospitalization, institutionalization and death. Bringing together researchers from seven European countries, the proposal aims to achieve the critical mass and the geographical extension enough to provide information useful to all older European citizens. An observational study will be carried out to calculate the incidence of the different outcomes in relation to the various interventions that will be assessed; results will be compared with data coming from already established national, regional and local dataset using the observed/expected approach. The sample will be made up by at least 2000 citizens for each outcome. All the citizens will be assessed at the baseline with two multidimensional questionnaires: the RISC questionnaire and the Short Functional Geriatric Evaluation questionnaire. The outcomes will be assessed every six-twelve months

## Introduction

Frailty is a multidimensional syndrome predisposing to the development of functional decline in older adults [1, 2]. It is characterised by a loss of physiological reserve, often in the setting of limited socio-economic resources that results in increased vulnerability to adverse healthcare outcomes. It is also associated with the increased use of social and health care services. Comprehensive frailty assessment facilitates the planning of health and social care services, both at an individual and population level [3–6]. This approach is only now beginning to be adopted in public health [6 – 8]. Even if assessment of frailty is not yet a common step for accessing appropriate care pathways, some European Union (EU) countries have developed integrated models of frailty assessment and good practices to address the management of chronic diseases that have been implemented locally or regionally in several member states [9–14].

To be successful in managing the care of frail older adults with chronic diseases, interventions must link health and social care systems together with supportive living environments capable of fostering the patient through different stages of diseases and functionality [15, 16]. The EU Commission and the Economic Policy Committee state that although increases in longevity must be accompanied by an increase in the number of years spent in good health and improvements in the health of those less well-off, this represents a policy challenge with potential significant repercussions on future expenditure trends [17]. A new public health approach, able to offer appropriate care to frail older patients through the different stages and severity of disease states, is therefore required. A pro-active model centered on frailty assessment [9] could become an entry point for patients and healthcare professionals to access integrated care, while the integrated management of chronic disease and frailty prevention programs could offer appropriate tailored care pathways to each patient [18,19].

While there are growing data suggesting that community-based programs assessing frailty and implementing interventions to prevent or mitigate frailty are able to impact upon its incidence and progression [9, 20], few studies have brought together data from several sources and researchers from different settings to investigate the scalability of differing approaches across the EU. This is a missed opportunity to assess crucial information that may influence healthcare policy and direct future funding. Indeed, it could be argued that inequalities across European countries and within each country are deep.

To pool, synthesize and evaluate many of the ongoing EU frailty studies represents an opportunity to take advantage of these often well-conceived but seemingly disparate projects. This approach is highly relevant given the emphasis the EU has been placing on preventing onset of frailty and functional decline through the launch of the European Innovation Partnership on Active and Healthy Ageing (EIP-AHA) and in particular, its A3 Action Group (EIP - AHA, DG Santé and DG CONNECT) [21]. The A3 Action Plan focuses on innovative approaches to prevent and manage frailty that include the fundamental components of education, training and empowerment of professionals and citizens [22]. It is also coherent with proposed work of the recently funded Joint Action on Frailty called ADVANTAGE, which plans to outline a joint framework to tackle frailty by tailoring available good practices to specific loco regional settings, thus facilitating scalability and adoption.

This paper describes the collaborative work (“synergy”): Impact of Community-based Program on Frailty Prevention and Mitigation of Frailty (ICP – PMF), launched by A1, A3, and B3 partners of the EIP - AHA, to investigate if common approaches to mitigate frailty in community dwelling older adults can be scaled-up specifically to develop and implement a practical screening approach to allow early detection of frailty. Thus, under the framework of the EIP-AHA’s Task Force on Synergies, a consortium of stakeholders from several EU countries (Table 1), led by the Biomedicine and Prevention Department of the University of Rome “Tor Vergata”, will measure the impact of several ongoing community-based interventions on the prevention and/or mitigation of frailty in line with the Monitoring and Assessment Framework for the European Innovation Partnership (MAFEIP) [23, 24].

The ICP –PMF synergy proposes to develop a feasible, achievable and manageable project using existing networks and stakeholders committed to the A3 Action Plan of the EIP –AHA [21,22] i.e. those currently involved in ongoing commitments (funded and unfunded projects) of the EIP- AHA. (Table 2).

### Operational definition of frailty for the ICP – PMF synergy

At present, no standardised operational definition of frailty is accepted [25–27]. The international debate has focused on two main approaches to defining frailty: the first one addresses physical determinants (physical definition), while the second one takes into account cognitive, nutritional, psychological and socioeconomic factors (bio-psycho-social definition). For the purpose of this study the synergy consortium selected the latter definition given that that is the most commonly used by the partners, as well as the more appropriate in the framework a public health approach aimed at mitigating frailty [15, 28, 29].

In this sense, the operative definition of frailty relates to the risk of adverse healthcare outcomes (disability, hospitalisation, institutionalisation and death) to which the individual is exposed given the association between frailty level and risk: the higher the frailty level, the higher the risk [30, 31]. The incidence of these outcomes relates not only to the patient’s functional, physical or mental status but also to their socioeconomic status. Lacking social and/or economic resources leads to an increased use of acute care or long-term care services even if the individual has minimal functional impairment [32, 33].

**Figure 1:**
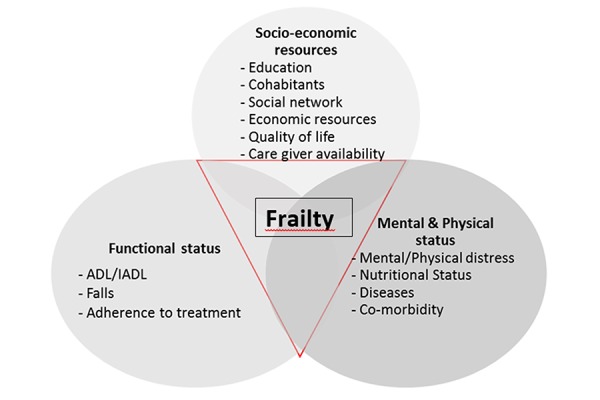
Characteristic of frailty according to its domains and indicators.

It is well established that the risk of death is associated with social isolation and that a strong social network has a protective effect (34]. For these reasons, the role played by socio-economic resources in determining the incidence and prevalence of frailty needs consideration (see Figure 1).

## Objectives of the ICP –PMF Synergy proposal

### General Objectives

- To set up a public health approach to prevent, identify and manage frailty in community dwelling older adults that can be validated in different EU member states.
- To identify factors that should be targeted in order to delay or postpone further decline and disability.

### Specific objectives

- Emphasize the importance of comprehensive frailty assessment as good practice in the prevention and management of frailty by counteracting social isolation, improving nutrition, promoting adherence to therapy and encouraging physical activity.
- Promote the continuum of care by integrating social and health care at primary, secondary levels.
- Assess the impact of this public health model on the management of frailty in the community in terms of cost effectiveness, use of health services and acceptance by citizens.
- Test the relationship between a set of indicators and the prevalence of frailty
- Investigate the readiness for ICT supported management
- Describe the strengths and weaknesses of caregiver network’s, their role in the management of frailty, while implementing strategies to maintain, supplement and improve this network.

## Methodology of the ICP–PMF Synergy

### Study design

Prospective observational study. The results of the observation will be analyzed according to the observed/expected approach (indirect standardized ratio) with the same outcomes observed in the population matching the study population for age and sex distribution, and location of residence. The analysis will consider three approaches in order to compare the outcomes: a) pooling all together the citizens who underwent whatever intervention vs the ones who did not undergo interventions; b) compare who underwent both social and health intervention vs the ones who receive only social or health intervention and vs the ones who did not undergo intervention; c) assess the impact of specific intervention against a control group set up specifically for this purpose.

The sources of the comparison will be mainly data already available from established data-flow at international, national and local level. To give an example data on hospitalizations are largely available at national and local level and could represent the standard of care against the outcomes of the planned intervention could be compared. Studies utilizing this kind of data, carried out by stakeholders involved in this proposal are ongoing [35–36].

### Data collection

All the study sites will perform a baseline assessment with the instruments selected in the preparation phase and a biannual/annual assessment of the outcomes.^1^.

### Outcomes

Each site in the study will assess at least one of the following outcomes:

- Incidence of frailty^2^ (e.g. surrogate markers including ADL/IADL impairment and/or reducing socio-economic resources and/or risk of adverse healthcare outcomes such hospitalization, institutionalization, death)
- Progression/regression of risk of negative outcomes
- Incidence of hospitalization
- Incidence of institutionalization
- Incidence of death

Each study site may add different outcomes listed in the specific study site section

### Sample

Subjects for inclusion/assessment will be aged over 64 years and living at home; those living in an institution (nursing homes or similar) will be excluded. Each study site can decide to restrict the field of intervention with additional exclusion criteria. The total number of European citizens involved in the assessment will be around 2000 for each outcome of which at least 20% will be enrolled before the end of June 2016.

Participants will be enrolled among the ones who access the services managed by the stakeholders involved in the proposal. In Ireland, participation in the study will be offered to community-dwelling older adults who are under surveillance by their Public Health Nurse consistent with ongoing studies in this setting [10, 13, 14]; in Valencia participant will be the women who access the outpatients department of the University Hospital gynecologic service for health control. In Genoa participants to the DOREMI project will be enrolled enrolled involving citizens who access to the local University Hospital. In Barcelona participants will be enrolled among communitydwelling older adults who are attended by their primary health care team, both in the office and at home. In Twente and Campania, community dwelling older adults will be enrolled among participants to the PERSSILAA project, patients referring to Federico II outpatients practices and to the GPs of the Salute in Collina no profit organization. Campania study sample will also include participant to the Sunfrail pilot, and to the Beyond Silos project in Salerno. In Rome the over-74 citizens reached by the Viva gli Anziani program (all living in the community) will be included in the study. In Porto participants will be primary care patients of Health Centers that agreed to participate. People over 64 years will be prescreened by trained GPs and further assessed by the CARTS project team, supported by the General Directorate of Health. In Parma the over 64 citizens of Central District of Parma will be included as part of Sunfrail and SprinTT recruitment process. In Pecs community dwelling older adults will be enrolled from members and clients of local NGO’s Wherever possible the General Practitioner of the citizens involved in the study will be informed.

### Sample size

– Alpha error = 5%
– Power = 80%
– Confidence Level = 95%
– Confidence Interval = 1%
– Population = 28,737,910^3^
– Expected reduction of frailty incidence = 2.5%
– Expected reduction of hospitalization rate = 5%
– Expected reduction of Institutionalization rate = 5%
– Expected reduction of death rate = 2.5%
– Test 1 Proportion (observational study) superiority: sample size needed per single outcome = 2,000 subjects (including 10% of lost-to-follow up)^4^
– Comparisons will be carried by applying the observed/expected approach on the basis of data stemming from the existing data flows available in the different study sites (national, regional or local data flow). The stratification of risk carried out by administering the baseline assessment will allow also transnational comparisons

### Study sites

The proposal includes 12 implementation sites located in seven EU countries (Table 3). However the characteristic of the proposal is to be open to other contribution by other sites/stakeholders who will make themselves available to participate to the study

### Baseline assessment

The baseline assessment aims to stratify the population, (from robust to frail individuals) according to the risk on negative outcomes. The basis of the stratification is the operative definition of frailty above-mentioned.

The tool to be used should:

- Identify frail individuals and size the risk of hospitalization, institutionalization and death as well as the trend of this risk during the follow up according to the exposure to interventions to cope with frailty
- Cover the five domains of frailty: social and economic resources, functional status, physical and mental health;
- Be short and easy to administer in order to involve thousands of citizens;
- Be simple in order to involve non –health professional personnel, such as the care-giver to administer it.

The working group reached a consensus on two common instruments for baseline assessment:

The **Risk Instrument for Screening in the Community** can be used to quickly screen large numbers of patients to identify and stratify those at greatest risk of three adverse healthcare outcomes (institutionalisation, hospitalisation and death) [10, 13, 37–41]. These patients can then be triaged for further assessment, investigation and treatment with integrated care bundles or other management strategies that are location or service specific. The RISC tool collects demographics, records concerns, the severity and ability of the caregiver network to manage three main domains (mental state, ADL state and medical state issues). It then summarises the perceived risk using a subjective, global score of risk based upon a five-point Likert scale measured from 1 (minimal-rare risk) to 5 (extreme-certain). The RISC was developed as an exemplar under the EIP on AHA reference site COLLAGE, Ireland’s only reference site for active and healthy ageing. It has been translated into multiple languages (English, Dutch, Italian, Spanish and Portuguese). It has been validated in multiple sites in the EU as part of work conducted in Action Group A3 of the EIP on AHA including in Porto (34) and Barcelona. The RISC stratifies risk of adverse healthcare outcomes by measuring the magnitude of functional, physical or state mental concern.

Sometimes although the concern may be minimal, the lack of formal and/or informal caregivers may elevate or even multiple that risk.

In order to better explore these borderline patients there is a need also to include a second instrument, aimed at assessing the risk of negative outcomes in individuals with no or minimal physical and/or cognitive impairment. **The Short Functional Geriatric Evaluation** is the synthesis of the Functional Geriatric Evaluation (FGE) questionnaire already validated for the predicting of negative outcomes within 5 years from the administration in an Italian population. The SFGE shows good correlation with the FGE [30,42] (Spearman Correlation = 0.82, p<0.001 tested on 203 individuals). This proposal will assess the harmonization of this procedure for the common baseline assessment with the different procedures developed by the projects embedded in the proposal in order to maximize the quantity and quality of information on the effectiveness of the implemented approaches.

### Follow up

**Figure 2:**
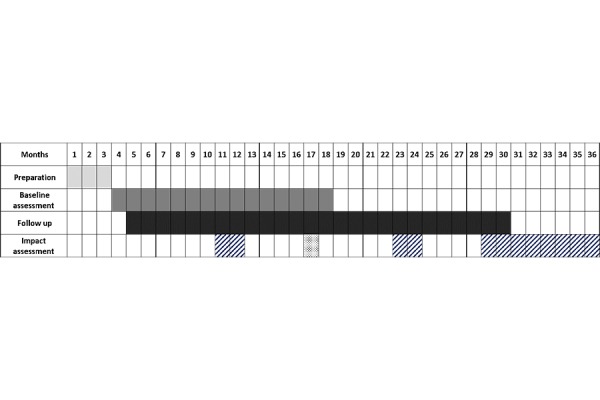
the GANNT Chart: Start date: January 1^st^ 2016.

According to the different program/projects intervention schemes, follow up data will be gathered every six-twelve months. The follow up will consist of the information that will be obtained following the baseline assessment on incidence of frailty, hospitalization, institutionalization and death. The study will run for 36 months in total including three months set up time and three months at the end to finalize the assessment (Fig 2)

Table 3 reports the list of study site with the list of data sources to be considered in the study for the comparisons. In some cases different kind of control groups will be also used: historical or ongoing cohort of citizens, assessed for frailty with comparable tools, who did not underwent any intervention will be involved in the data analysis. Data will be analyzed every six months

### Ethical consideration

Each study site will ensure ethical approval for each component of the study, according to local regulation. The study has already been approved by the following institutions: the Independent Ethic Committee of the University of Rome “Tor Vergata”; the Ethic Committee of the “Federico II” University of Naples, the Ethics Committee of the University Hospital in Valencia; the Medical Ethical Committee Twente; the IDIAP Jordi Gol Clinical Research Ethics Committee; the Regional Ethic Committee of Liguria; the ethics committee of the Regional Association of Health North (ARS North), and by each of the Associations of Health Centers in the region where data will be collected in Porto.

### Budget and available resources

Most of the activities encompassed by the synergy are already financed and will go on for the next three years; further resources will come from each individual study site as work in-kind, utilising internal resources (e.g.printing materials and consumables). Additional budget, especially to support the ICT infrastructure and to scale up and maintain services is currently not foreseen and is being sought.

## Development of the program

The initial development of the ICP-PFM proposal over the first 12 months, is reported in Table 4: the first two sprints have already been achieved by 30 June 2016: a synthetic report on baseline assessment is in progress. The S4 sprint will be available by the end of 2016; it deal with the impact of the different interventions put in the field. This is a realistic picture of the aim of the whole proposal: to provide data about the effectiveness of Community-based intervention focused on frail citizens to reduce the negative outcomes of frailty itself and to contribute to both the sustainability of the health and social system and the equality of care opportunities (or at least procedures) throughout the EU countries.

## Conclusion

The synergy proposal is the result of sharing a pathway: assess the impact of community-based programs on the healthy and the use of services of community-dwelling older adults in order to select what is effective in terms of both reduction of costs for the providers of health services and negative outcomes for the individuals. The main goal is to scale-up of common approaches to mitigate the impact of frailty on community-dwelling older adults, by implementing a practical screening approach that allows early detection of frailty followed by strategies to manage frailty and functional decline. These approaches should reduce the incidence of adverse health outcomes in EU older citizens, who should improve their quality of life, and at the same time make health systems more efficient and sustainable. Finally these approaches include the implementation of new professionals and/or new models of care (including the implementation of ICT solution for better life) which encompass the collaboration among formal and informal carers: this is one way towards creating new opportunities for business that is the third component of the “triple win” boosted by the EIP-AHA [43].

## Figures and Tables

**Table 1:** Composition of the Impact of a Community-based Program on Prevention and Mitigation of Frailty

Leading organization	Biomedicine and Prevention Department, University of Tor Vergata,		Rome	Italy
Supporting organization	University College Cork, National University of Ireland, Galway	CARTS program AGs A1, A3	Cork	Ireland
			Galway	Ireland
	IDIAP Jordi Gol,		Barcelona	Spain
	UNIFAI/ICBAS-University of Porto		Oporto	Portugal
	Community of Sant’Egidio	Long Live the Elderly Program AG A3	Rome	Italy
	“Federico II” University Hospital	PERSSILAA and SIMPATHY projects AGs A1, A3	Naples	Italy
	LUMSA University		Rome	Italy
	Medical University of Graz		Graz	Austria
	Educators’ Centre Association		Pecs	Hungary
	SI4LIFE scrl	DOREMI project AG A3	Genoa	Italy
	University of Parma – Emilia Romagna Region	SUNFRAIL project AG – A3	Parma	Italy
	University of Twente	PERSSILAA project AG A3	Twente	The Netherlands
	University of Salerno	Beyond Silos AG – B3	Salerno	Italy
	University of Valencia	FOCUS Project AG - A3	Valencia	Spain

(ICP –PMF) synergy consortium.

**Table 2:** European projects embedded within the synergy proposal.

Title of the project	Acronym	DG	Programme	Starting date	End date	Budget (M€)*
Mechanisms of the Development of Allergy	MeDALL	R&D	FP7	12-2010	06-2015	16 (12)
PERsonalized ICT Supported Service for Independent Living and Active Ageing	PERSSILA A	R&D	FP7	11-2013	11-2016	3.2 (2.5)
Citizen Reinforcing Open Smart Synergies	CROSS	ICT PSP	CIP	12-2012	06-2016	5.4(2.7)
Stimulating Innovation Management of Polipharmacy and Adherence in the Elderly	Simpathy	SANTE	3rd Health Program 2014–2020	06-2015	05-2017	1.2 (1)
Beyond silos – Learning from integrated eCare practice and promoting deployment in European regions	Beyond Silos	Enterprise and Industry	CIP ICT Policy Support Programme	02-2014	01-2017	5.4 (2.4)
Reference Sites Network for Prevention and Care of Frailty and Chronic Conditions in community dwelling persons of EU Countries	Sunfrail	SANTE	3rd Health Program 2014–2020	05-2015	10-2017	1.6 (0.8)
Frailty management Optimisation though EIP AHA Commitments and Utilisation of Stakeholders input	FOCUS	SANTE	PUBLIC HEALTH PROGRAM	05-2015	04-2018	2.5
Decrease of cOgnitive decline, malnutRition and sedEntariness by elderly empowerment in lifestyle Management and social Inclusion	DOREMI	CONNECT	FP7	11-2013	11-2016	3,7(2,9)

* Total budget, in brackets: the budget obtained from the EU

**Table 3:** summary of the study design according to the implementation sites

Study site	Population^5^	Study design	Tools	Activities	Outcomes	Source(s) of comparison
Barcelona	Over 64 (1000 ind.s)	Prospective observational	-SFGE - RISC	-Frailty Pro-active screening/stratification and monitoring of community dwellers. -Referral to formal community-based or hospital-based services in case of need. - Community-based social activities including health promotion - Psychoeducational programmes for informal caregivers -Introduction of harmonised care bundles addressing multimorbidity, cognitive function and nutrition	Incidence of hospitalization Incidence of institutionalization Incidence of death	Official sources Data from different representative elderly cohorts in Catalonia, CARTS project in Cork, Porto and Galway. data stemming from the same sample according to a before-after interrupted-time series design
Cork	Over 64 (5000 ind.s)	- Prospective observational - Randomised trial (pilot)	-CFS -CARTS -RISC -SFGE	-Frailty screening/stratification -Targeted intervention	Incidence of frailty Progression of frailty Incidence of hospitalization Incidence of institutionalization Incidence of death	CARTS project in Galway
Galway	Over 64 1000 ind.s	Prospective observational	-CFS -CARTS -RISC -SFGE	-Pro-active frailty screening/stratification and monitoring -Introduction of a harmonised care bundle, common to the community-hospital interface (i.e. an integrated care pathway). -Referral to formal community-based or hospital-based services in case of need.	Progression of frailty Incidence of hospitalization Incidence of institutionalization Incidence of death	The CARTS project in Cork as a sister study. Retrospective data on older adults attending University Hospital Galway during the corresponding period one year before the interrupted time series study commences.
**Genova**	Over 64 (about 30 Individuals)	Prospective observational Randomised trial (pilot)	-SFGE -RISC	In particular, DOREMI activities include: early risk detection of : ○ malnutrition ○ cognitive decline ○ social isolation ○ sedentariness ○ falls early risk ICT intervention based on social nudges and gamification to the promote active and healthy lifestyle and behavioral in respect to the four main risk factors : change and maintain healthy nutrition lifestyle reduce social isolation change and maintain an healthy physical lifestyle train cognitive function and reduce cognitive decline	Progression of frailty Incidence of hospitalization Incidence of institutionalization Incidence of death	a) Official sources (report on the hospitalization and mortality in Italy and UK released annually by EUROSTAT, ISTAT etc.); b) Passi d’Argento report (Regione Liguria) b) Other sources (data stems from the ongoing project).
Naples	Over 74 (175 ind.s)	Prospective observational	-SFGE -RISC	-Pro-active frailty screening/monitoring -Assistance in bureaucratic tasks -Referral to formal community-based or hospital-based services -Strengthening the social involvement of pre-frail older adults -Community-based health and ICT literacy activities including health promotion -Introduction of assessment of polypharmacy regimen	Incidence of frailty Progression of frailty Incidence of hospitalization Incidence of institutionalization Incidence of death	Official sources (report on the hospitalization and mortality in Campania region released annually by ISTAT and Ministry of Health); data stems from the ongoing Lazio Region Frailty Study carried out by the University of Tor Vergata, Regional pharmaceutical Data flows (evaluation of polypharmacy)
Parma	Over 64 (1000 ind.s)	Prospective observational	-Sunfrail -SFGE -RISC	-Pro-active frailty screening of 1000 over-65 citizens followed by comprehensive geriatric assessments -Referral to day services or day-hospital-based services in case of need -Strengthening the social network around the frails -Activation of nutritional, physical exercise and technological monitoring interventions with specific targets: -Prevention of falls -Prevention of negative consequences of social isolation -Prevention of Malnutrition	Incidence of frailty Progression of frailty Incidence of hospitalization Incidence of institutionalization Incidence of death	Official sources (report on the hospitalization and mortality in Emilia Romagna region released annually by ISTAT and Ministry of Health);
Pecs	Over 64 (200 ind.s)	Prospective observational	-SFGE -RISC	-Frailty screening -training of the target group) on ICT skills -training of informal caregiver network on the use novel assessment and training tools. -widespan public awareness raising campaign of the general public in all participating countries	Incidence of hospitalization Incidence of institutionalization Incidence of death	official statistical data of the National Health Insurance and Statistical Bureau
Oporto	Over 64 (300 Ind.s)	Prospective double arm observational	- RISC	-A training program for PC professionals on frailty and dementia screening and intervention -A awareness program for the general community on frailty and dementia issues. -A psycho-educational program for caregivers of old people frail or with dementia. -A cognitive stimulation program for people with cognitive frailty or MCI	Incidence of hospitalization Incidence of institutionalization Incidence of death	Control group
Rome	Over 74 (4500 ind.s)	Prospective observational	-SFGE -RISC	-Pro-active frailty screening/monitoring -Home visits -Assistance in bureaucratic tasks -Referral to formal community-based or hospital-based services in case of need -Strengthening the social network around the frails -Community-based social activities including health promotion with specific targets	Incidence of hospitalization Incidence of institutionalization Incidence of death	Official sources (report on the hospitalization and mortality in Campania region released annually by ISTAT and Ministry of Health); data stems from the ongoing Lazio Region Frailty Study carried out by the University of Tor Vergata,
Salerno	Over 64 (100 ind.s)	two arms prospective observational	-SFGE -RISC	-Multidimensional assessment -Social and Health care integration -Electronic Clinical Record filled by the personnel and by the patient/caregiver -Home care monitoring and treatment by remote -Clinical management	Incidence of hospitalization Incidence of institutionalization Incidence of deaths	Control group
Twente	65–75 (300 ind.s)	two arms prospective observational	-GFI -SF-12 -SF-36 -MNA -SFGE -QMCI -NMA -Phys. test^6^	-frailty screening through multidimensional assessment -The PERSSILAA physical module Web-based exercising Monitoring	Incidence of frailty/functional decline Quality of LIfe	Control group
Valencia	Over 64 (1600 women)	Prospective observational	- Fried - CFS - SFGE - RISC	-Clinical assessments -Analytical Lab assessments -Bone metabolism assessment -Frailty assessment. -Cognitive frailty -Quality of life -Lifestyle intervention	Incidence of frailty Progression of frailty Incidence of hospitalization Incidence of institutionalization Incidence of death	pre and post-intervention assessment comparison with other cohorts within the working group (Twente)

**Table 4:** SPRINTS forecasted by the synergy proposal

N∘	Name	Starting date	Delivery date	Geographic coverage	Results
**S 1**	Set up a community - based public health approach to scale-up multidimensional screening tools in order to prevent, detect and manage frailty in community dwelling elderly. Target population and procedure to deliver the screening will be agreed. Ongoing interventions will be included into a common pathway defined by the assessment methodology.	15-Jan-2016	31-Mar-2016	Six Countries	Report on the action plan to implement a public health approach to frailty in community dwelling elderly and on the assessment methodology
**S 2**	Set up of a methodology to find out the impact of the public health program in this field It will be based on the agreement about the use of one methodology to assess frailty at baseline 12 and 24 months of observation (main candidate the RISC questionnaire – CARTS program), and to identifying common outcomes to be evaluated after 12/24 months of observation. Data will be gathered into one database that will be the main source of the evaluation. Comparative data will stem from official data source (mortality data, institutionalization rate, hospitalization rate, cost of services) which encompass the studied population. Specific observational cohorts could be included in local trials aimed to assess the impact of the interventions: this kind of approach could take longer than the first three months to be set up. Additional tools to assess specific aspects of the projects supporting the proposal, which are already used, could be included to improve the assessment methodology.	15-Jan-2016	31-Mar-2016	Six Countries	
**S 3**	Risk Classification of the first 20% of the sample About 200–400 citizens	1-Apr-2016	30-Jun-2016	Six countries	Report on the baseline assessment
**S 4**	First six months Impact assessment	1-Nov-2016	31-Dec-2016	Six countries	Report on the impact assessment on the citizens assessed before the 30 June 2016

## Footnotes

1 In some study sites the inclusion of a prospective observational control group is already forecasted. This approach may offer advantages in terms of comparison, especially if the control group has been assessed for frailty with the same or correlated instruments implemented also in the present assessment.

2 Standardized ratio of frailty incidence are largely unknown. In this case the assessment will provide absolute risk of frailty unless a control group is followed up

3 Sum of the 2014 over-64 population living in Italy, Ireland, Portugal, Spain, The Netherlands and Hungary. Source: Eurostat

4 http://www.openepi.com/SampleSize/SSPropor.htm

5 Maximum number of individuals who can be potentially involved in the observational study per site

6 Two minute step test, time up and go test, sit to stand test, sit and reach test
